# A comparison between hypertonic dextrose prolotherapy and conventional physiotherapy in patients with knee osteoarthritis

**DOI:** 10.3892/mi.2023.105

**Published:** 2023-08-29

**Authors:** Kamil Mursit Yildiz, Hayal Guler, Halil Ogut, Mustafa Turgut Yildizgoren, Ayse Dicle Turhanoglu

**Affiliations:** 1 Department of Physical Medicine and Rehabilitation, Medical School, Hatay Mustafa Kemal University, Antakya, Hatay, 31001, Turkey; 2 Department of Physical Medicine and Rehabilitation, Konya City Hospital, Karatay, Konya 42020, Turkey

**Keywords:** isokinetic test, knee osteoarthritis, knee pain, physiotherapy, prolotherapy

## Abstract

The aim of the present study was to compare the efficacy of hypertonic dextrose prolotherapy (HDP) with conventional physiotherapy (CPT) in improving symptoms in females with knee osteoarthritis (OA). The present study included 60 patients with a diagnosis of knee OA. The patients were randomly assigned to the HDP (n=30) and CPT (n=30) groups. The patients in the HDP group were treated with a dextrose injection into the knee joint (25% dextrose) and around the knee (15% dextrose) in two sessions for 1 month, while those in the CPT group received a hot pack, transcutaneous electrical nerve stimulation and therapeutic ultrasound in five sessions a week for 4 weeks. Prior to commencing the treatment, and at 1 and 3 months post-treatment, all the patients were evaluated using the visual analog scale (VAS), Western Ontario and McMaster Osteoarthritis Index (WOMAC), the goniometric measurement of active knee range of motion (ROM), a 50-m walking test and isokinetic knee muscle strength measurements. There were no statistically significant differences between the two groups as regards the demographic characteristics at pre-treatment (P>0.05). However, at 1 and 3 months post-treatment, the scores of all the outcome parameters were significantly improved in the HDP group compared with the CPT group (P<0.05 for all). In both groups, a significant improvement was observed in the VAS scores, WOMAC total values and ROM following the treatments, with the greatest improvement observed in the HDP group (P<0.001). The isokinetic quadriceps peak torque measurements were increased in both groups following treatment. All the scores exhibited a statistically significant improvement in the HDP group at both 1 and 3 months post-treatment. On the whole, the results of the present study demonstrate that both HDP and CPT are effective treatment modalities to relieve pain, and increase functionality and strength in patients with knee OA. However, greater improvements in pain and functionality can be achieved with prolotherapy.

## Introduction

The chronic and progressive nature of knee osteoarthritis (OA) often results in a poor quality of life, and the disability created by this joint disease creates a significant social and economic burden for both patients and caregivers (1). In the past, OA was only considered to be a degenerative joint disease; however, recent research has demonstrated a more complex pathogenesis, which has encouraged the development of new treatment strategies. There is currently no cure for OA, and the available treatments mainly focus on symptom relief and improving disability rather than halting progression of the disease (2). For optimal management of knee OA, a combination of non-pharmacological and pharmacological modalities is required, and surgery is recommended for intractable cases (3). The pharmacological and non-pharmacological treatment options for the management of knee OA include changes to daily living activities, weight loss through dietary interventions and exercise, manual therapy, physical therapy, electrotherapy [transcutaneous electrical nerve stimulation (TENS), therapeutic ultrasound (US) and the use of lasers], taping, the use of assistive devices, bracing, non-steroidal anti-inflammatory drugs, acetaminophen, opioids and injection therapies (dextrose prolotherapy, ozone, platelet-rich plasma and hyaluronic acid) (4,5).

Prolotherapy is as a non-surgical regenerative technique, in which small amounts of an irritant solution are injected to the site of painful or degenerated tendon insertions, joints, ligaments and adjacent joint spaces, with the aim of promoting normal cell and tissue growth (6). Hypertonic dextrose at concentrations of 12.5 to 25% is the most widely used prolotherapy solution, and multiple clinical trials have reported favorable outcomes. For several decades, musculoskeletal pain has been treated with intra-articular or extra-articular injections of dextrose infiltration over ligament and tendon insertions. Previous controlled trials have reported that these treatments with hypertonic dextrose prolotherapy (HDP) are effective in patients with symptomatic knee degeneration. For example, in a previous randomized controlled trial, 90 adults who had been experiencing painful knee OA for a minimum of 3 months were randomly assigned to one of three groups: Blinded prolotherapy, saline injections, or at-home exercise. The participants were then followed-up with visits at the 52-week mark. Upon follow-up, individuals who underwent dextrose prolotherapy exhibited better WOMAC scores compared to those who received saline injections or engaged in at-home exercise. Moreover, the patients who received dextrose prolotherapy reported high levels of satisfaction (7). Reeves and Hassanein (8) conducted a study involving patients with knee laxity who underwent dextrose prolotherapy treatment. Their findings revealed that following a 12-month follow-up period, the group receiving the treatment exhibited notable enhancement in laxity when compared to the control group (8). Although the mechanisms of prolotherapy are not yet fully understood, some researchers have provided results supporting the theory that HDP injection triggers an inflammatory cascade following cell shrinkage, which then increases the release of collagen deposition and growth factors (9). The aim of the present study was to compare the efficacy of HDP with conventional physiotherapy (CPT) in improving pain, movement restriction, walking speed, activities of daily living and isokinetic muscle performance in female patients with knee OA.

## Patients and methods

### Study participants

The present study with a randomized prospective study which included a total of 60 female patients with a confirmed diagnosis of knee OA in accordance with the Kellgren-Lawrence criteria (10), who were referred to the PM&R outpatient clinic of Hatay Mustafa Kemal University Medical School (Antakya, Turkey) between July, 2020 and December, 2021. The main inclusion criterion was the radiographically confirmed presence of mechanical knee pain, around the knee joint, which had been ongoing for at least 3 months. The study exclusion criteria were defined as an age <50 years, the presence of an inflammatory rheumatological disease, grade 1 or 4 OA based on the Kellgren-Lawrence radiological criteria, a history of knee surgery or joint replacement, trauma, any intra-articular injection (hyaluronic acid, steroids or platelet-rich plasma) over the past 6 months, malignancy, or any other neurological disorder that could contribute to the symptoms. Approval for the present study was granted by the Medical Ethics Committee of Hatay Mustafa Kemal University (decision no. 2020/75). Written informed consent was provided by all the study participants. The present study was registered in the ClinicalTrials.gov database (NCT04958213).

**Figure 1:**
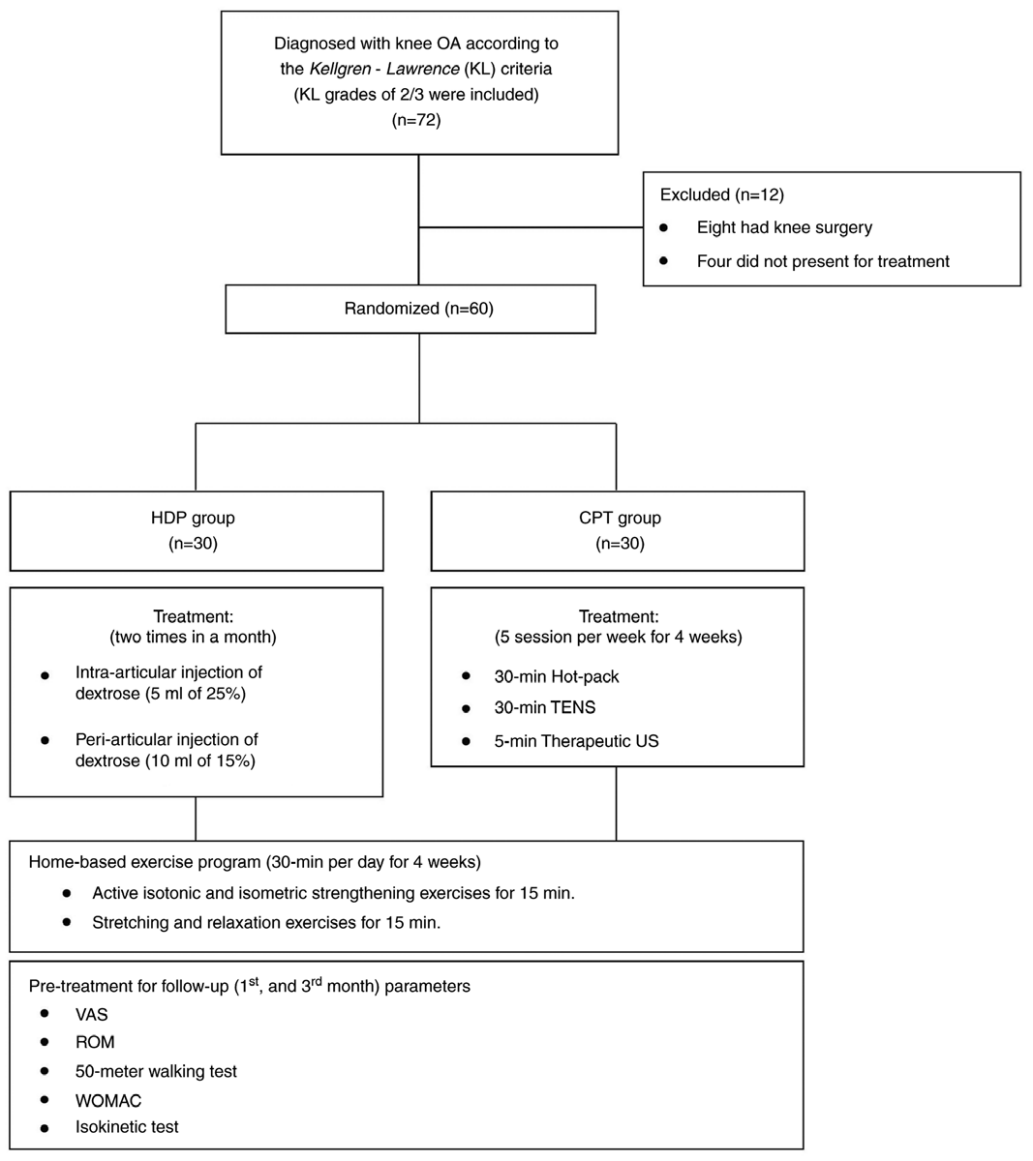
Flowchart of the selection process for the patients in the present study.

The patients were assigned to the HDP or CPT group using a simple randomization method using a table of random numbers, assigning 30 patients to each group. All the patients had been recommended to perform knee exercises for 1 month. Throughout the study period, the patients were requested not to take any painkillers, but were permitted to take paracetamol if deemed necessary. The study flowchart is presented in Fig. 1.

### HDP

**Figure 2:**
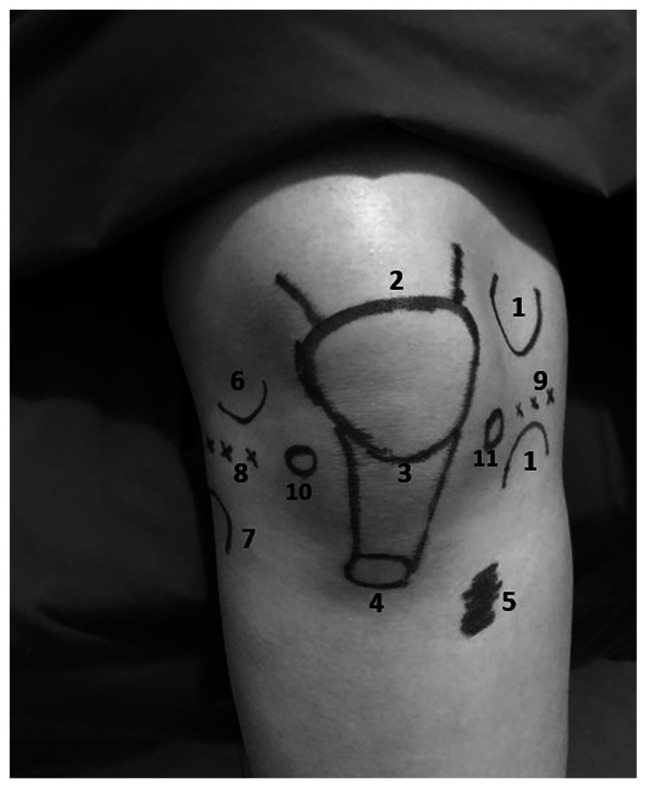
The figure shows schematically possible prolotherapy points in a healthy model. The injection sites used for hypertonic dextrose prolotherapy. 1, proximal and distal medial collateral ligament; 2, quadriceps tendon region of the patella upper edge; 3, proximal region of the patellar tendon; 4, distal of the patellar tendon; 5, tendon region of pes anserine; 6, proximal lateral collateral ligament; 7, distal lateral collateral ligament; 8, lateral coronary ligament; 9, medial coronary ligament; 10 and 11, intra-articular injection sites.

The procedure was performed by a qualified physical medicine and rehabilitation physician. With the patient placed in the supine position, and the knee was placed at 20-30˚ flexion, the injection area on the lateral side of the knee was identified. Using a 27-G needle, aspiration and correct needle placement in the joint were ensured, and the injection was then performed. In the HDP group, all the patients received an intra-articular injection of 5 ml 25% dextrose (2.5 ml 20% dextrose + 2.5 ml 30% dextrose), and a peri-articular injection of 10 ml 15% dextrose (5 ml 0.9% NaCl + 5 ml 30% dextrose) to each ligament-bone insertion. The injection sites were identified using anatomic landmarks; two injections were performed at a 2-week interval. The injection points were designated as the medial and lateral coronary ligaments, proximal and distal medial and lateral collateral ligaments, the quadriceps tendon region of the patella upper edge, and the distal and proximal region of the patellar tendon, and the tendon region of pes anserine (Fig. 2). In the HDP group, mild warmth and redness around the knee were observed for 3 days in 2 patients after the injection; however, this condition improved without any issues during the follow-up.

### CPT

In the CPT group, all the patients received combined hot pack (HP), US and TENS treatments. A physical therapy program was applied to patients in the CPT group 5 days a week for 4 weeks as a total of 20 sessions. Using a two-channel portable TENS unit (BTL-4620, BTL Corporate), TENS therapy was applied around the knee region for 30 min with two electrodes in conventional mode, at a frequency of 100 Hz and a pulse width of 60 msec and intensity adjusted according to the threshold for each patient without causing pain or muscular contraction. US sessions of 5 min continuously were performed 5 days a week for 4 weeks for a total of 20 sessions, using a power of 1 W/cm^2^, and frequency of 1 MHz (BTL-4000 Professional, BTL Corporate). HP therapy was applied for 30 min per session for a total of 20 sessions as a part of the conventional physiotherapy.

### Exercise

A home-based exercise program was performed by all the patients in both groups 5 days a week for 4 weeks. The program included active isotonic and isometric strengthening exercises for 15 min, and stretching and relaxation exercises for 15 min.

### Outcome evaluations

Before treatment, and at 1 and 3 months after the final injection, both groups completed the standard questionnaire including the Western Ontario and McMaster Universities Arthritis Index (WOMAC) (11), and visual analog scale (VAS).

The severity of pain felt in the knee was measured using the VAS scores ranging from 0 (no pain) to 10 (worst possible pain). The WOMAC scale was used to evaluate the functional status of the patients. WOMAC is a measure of performance that examines three categories of function, including pain (five items) and physical function (17 items). Each item is scored as follows: None, 0; mild, 1; moderate, 2; severe, 3; or very severe, 4, with lower scores indicating a better condition. Active knee joint range of movement (ROM) was measured using a manual universal goniometer, the 50-meter walking time, and the measurements of isokinetic knee extensor/flexor muscle peak torque (PT). These evaluations were made prior to treatment, and at 1 and 3 months post-treatment.

Isokinetic muscle strength was measured using the Humac^®^ NORM isokinetic dynamometer (Computer Sports Medicine Inc.). The extensor and flexor muscles of the affected knee of the patients in both groups were evaluated with isokinetic tests prior to treatment, and at 1 and 3 months post-treatment. Each patient was seated on the dynamometric chair and stabilized with waist and chest belts in a 90˚ sitting position for the isokinetic measurement. Following five submaximal warm-up contractions, an evaluation was made of the concentric PT values of the quadriceps and hamstring at 60 and 180˚ per second angular velocities. The protocol for the isokinetic test protocol was five repetitions at 60˚ per second, 30 sec of rest, and 15 repetitions at 18˚ per second.

### Statistical analysis

The data obtained in the present study were analyzed statistically using SPSS version 22.0 software (IBM Corp.). Descriptive statistical results are presented as the mean ± standard deviation (SD) values for continuous data, and as number and percentage for categorical data. Demographic data were analyzed using the independent samples t-test for continuous variables and the Chi-squared test for categorical variables. The differences in the scores of each group at the different times measured were analyzed using the repeated measures analysis of variance (ANOVA) test. Following repeated measures ANOVA, the paired t-test with the Bonferroni correction was used. Differences between the groups were compared using the independent samples t-test. A value of P<0.05 was considered to indicate a statistically significant difference.

The power of the study was calculated using the G*Power 3.1.9.4 program after the study. In the evaluation using the difference between two independent means, the power of the study was found to be 99%. The effect size was calculated as 1.26 using the post-treatment VAS parameter.

## Results

No statistically significant differences were determined between the groups as regards age, weight, height, body mass index, symptom duration, Kellgren-Lawrence grade, VAS at rest, knee ROM, the 50-m walking test, WOMAC and isokinetic muscle performance values (P>0.05 for all). The demographic, clinical, radiographic and isokinetic data are presented in Table I.

In both the HDP and CPT groups, there were statistically significant differences between pre- and post-treatment (at 1 and 3 months) in terms of the clinical assessments: VAS (P<0.001), ROM (P<0.001), WOMAC (P<0.001), 50-m walking test (P<0.001) and isokinetic parameters (P<0.001) (Tables II and III). Comparisons between the groups of the VAS, WOMAC and isokinetic parameters revealed significant differences between the HDP and the CPT groups as regards the VAS score and flexor PT (180˚/sec AV) at 1 and 3 months post-treatment, and in the 50-m walking test scores at 3 months post-treatment (Tables II and III). Significantly greater improvements were observed in the HDP group compared with the CPT group as regards the VAS, WOMAC and extensor PT (60˚/sec AV) at 1 and 3 months post-treatment Table IV).

## Discussion

The aim of the present study was to compare the efficacy of HDP and CPT in patients with knee OA. The results demonstrated that both groups achieved successful outcomes, as measured by the lower WOMAC and VAS scores, and increased knee ROM and muscle strength. The efficacy of HDP was found to be more prominent than that of CPT, as regards VAS, WOMAC and ROM. When the two groups were compared, it was found that at the end of the 1st month, HDP was more effective than CPT in terms of VAS scores and isokinetic parameters (flexor PT at 180˚/sec AV) and at the end of the 3rd month, HDP was found to be more effective CPT in three parameters (VAS, 50-m walking test and flexor PT at 180˚/sec AV).

It has been stated that the more common treatment, CPT, is safe and effective in cases of knee OA, particularly as regards reducing pain, improving function and developing muscle strength. The current therapy for rehabilitation of knee OA focuses on reducing pain and improving function and joint ROM (12). CPT management includes thermal modalities that decrease spasms and pain, and help to improve joint ROM, and electrotherapy, which includes TENS, US and exercise (13). According to a previous systematic review that evaluated the efficacy of CPT in knee OA, thermotherapy, electrotherapy and exercise therapy resulted in reduced pain and improved function (14). The results of the present study demonstrated that CPT had positive effects on a number of parameters in patients with knee OA at 1 and 3 months post-treatment. Previous randomized clinical trials of patients with knee OA have reported a greater efficacy of HDP in terms of pain relief and improvement in function compared with conservative treatments (physiotherapy or exercise programs) (15,16). The most likely reason for this effect is that HDP provides an analgesic effect based on both neurogenic mechanisms and through the repair of soft tissues and cartilage.

Prolotherapy is an injection therapy which is used in the treatment of chronic painful musculoskeletal conditions, including knee OA. The results of other randomized controlled trials, systematic reviews and meta-analyses have demonstrated an improvement in knee pain, function and stiffness scores in patients with knee OA of a moderate-to-severe degree (2,7,17-19). The standard injection protocol for prolotherapy includes a whole-joint approach with both intra-articular and extra-articular injections to the bony soft tissue attachments (20). In many studies that have investigated HDP used for the treatment of knee OA, the effectiveness has been compared with other treatments. A systematic review released in 2019 concurred that prolotherapy demonstrated superior effectiveness compared to local anesthetic infiltrations in terms of reducing pain and enhancing functional improvement (21). In addition, in that review, prolotherapy exhibited similarity to hyaluronic acid, ozone, or radiofrequency infiltrations, but showed lower efficacy compared to platelet-rich plasma (PRP) and erythropoietin over the short, medium, and long-term durations, according to available research (21). In another study, the intra-articular dextrose concentration used has ranged from 10 to 25%, with wide variations in the number of injections, break durations and follow-up periods (2). HDP has been shown to have a more beneficial effect than saline and home-based exercise therapies (7) and a similar effect to that of PRP in reducing pain (17). As it is simple to implement, the WOMAC scale is the most frequently used patient-reported outcome for knee OA. In the present study, both the HDP and CPT groups exhibited significant improvements in the WOMAC scores at 1 and 3 months post-treatment, compared to the baseline scores, with no apparent superiority of one treatment method over the other.

The currently proposed mechanisms of action are focused on the generation of low-grade inflammation related to the injection of hyperosmolar solutions. This process primarily relies on the generation of cytokines (22). At the fibro-osseous junction of ligaments and tendons, this inflammation leads to a healing cascade of various paracrine pathways relating to cell growth and repair. The direct needling of the tissue may also stimulate repair, with the disruption of cellular membranes and local blood supply resulting in the release of healing and inflammatory blood factors, such as calcitonin gene-related protein (CGRP), bradykinin and prostaglandins (23). The direct injection of hyperosmotic solutions, such as dextrose may also promote the activation of pain receptors, such as the capsaicin pain receptor. The upregulation of these channels results in an increase in substance P, CGRP and nitric oxide, which are considered to have a suppressive effect on receptors. In addition, the transmission of pain via the alpha-delta nerve fiber may result in endogenous opioid-mediated pain suppression, as described in the gate-control theory (22,23).

The evaluation of muscle performance with the isokinetic test is an objective method that is frequently used. The isokinetic test has been shown to be a valid and reliable measurement of the strength of the knee flexor and extensor muscles (24). Low angular velocity tests are more accurate in the measurement of muscle strength and high angular velocity tests are used to evaluate the muscle function and endurance (25,26). The tests used in the present study were 60˚/sec and 180˚/sec angular velocity. The presence of knee OA is known to reduce isometric knee strength and this will worsen with disease severity. Functional measurements have been shown to be associated with knee strength measurements performed with isokinetic dynamometers (27,28). Both groups in the present study exhibited improved results in the isokinetic test following treatment. Based on these findings, it can be ascertained that HDP and CPT may increase muscle strength in patients with knee OA.

There were several limitations to the present study. First, the present study did not examine whether the patients with OA performed the home-based exercise program regularly. Secondly, the patients were not questioned about their history of drug use. Thirdly, patients with mild and severe OA (Kellgren-Lawrence grade I and IV) were excluded from the study. Finally, the 3-month period of treatment may not have been sufficient for a definitive evaluation of muscle performance.

In conclusion, in light of the results of the present study, both HDP and CPT may be considered effective treatment modalities to reduce pain, and increase functionality and strength in patients with knee OA. However, prolotherapy was observed to have led to more notable improvements in pain and functionality.

## Figures and Tables

**Table I:** Demographic and clinical features of the groups of patients.

Parameter	HDP (n=30)	CPT (n=30)	P-value
Age, years	60.07±6.82	60.60±6.10	0.751
Weight, kg	81.07±13.52	77.60±8.66	0.242
Height, cm	159.23±5.38	158.40±5.34	0.550
Symptom duration, months	18 (1-240)	21 (6-48)	0.893
Body mass index, kg/m^2^	31.84±5.19	30.88±2.83	0.380
Kellgren-Lawrence, grade n (%)			
Grade 2	13 (43.3)	16 (53.3)	0.596
Grade 3	17 (56.7)	14 (46.7)	
Baseline evaluations			
VAS for pain	7.33±1.34	7.20±1.42	0.748
50 m walking test	52.30±6.32	54.10±6.83	0.294
Range of motion	123.33±3.77	123.53±3.36	0.829
WOMAC, total scores	59.83±11.23	60.70±10.45	0.758
Isokinetic evaluation, PT (Nm)			
60˚/sec AV - extensor	43.37±16.63	39.63±17.49	0.400
60˚/sec AV - flexor	17.57±10.29	21.90±13.01	0.158
180˚/sec AV - extensor	29.27±9.25	30.27±10.66	0.699
180˚/sec AV - flexor	11.67±6.84	19.90±9.60	**0.001**

Values in bold font indicate a statistically significant difference (P<0.05). HDP, hypertonic dextrose prolotherapy; CPT, conventional physiotherapy; VAS, visual analog scale; WOMAC, Western Ontario and McMaster Osteoarthritis Index; PT, peak torque; AV, angular velocity.

**Table II:** Baseline and after treatment (at 1 and 3 months) follow-up results of the clinical measurements of the groups.

Parameter	HDP (n=30)	CPT (n=30)	P-value
	Mean ± SD		
VAS for pain			
Baseline	7.33±1.34	7.20±1.42	0.748
After treatment			
1st month	4.47±1.77^a^	5.60±1.22^a^	**0.006**
3rd month	2.43±1.85^a,b^	4.40±1.03^a,b^	**0.001**
Knee ROM (degree)			
Baseline	123.33±3.77	123.53±3.36	0.829
After treatment			
1st month	124.43±3.66^a^	124.50±3.40^a^	0.942
3rd month	126.20±3.47^a,b^	125.60±3.50^a,b^	0.508
WOMAC (total score)			
Baseline	59.83±11.23	60.70±10.45	0.758
After treatment			
1st month	55.77±11.35^a^	58.20±10.78^a^	0.398
3rd month	51.93±11.13^a,b^	55.93±10.84^a,b^	0.164
50-m walking test (sec)			
Baseline	52.30±6.32	54.10±6.83	0.294
After treatment			
1st month	49.57±6.06^a^	52.07±6.77^a^	0.137
3rd month	46.97±6.23^a,b^	50.40±6.79^a,b^	**0.046**

Values in bold font indicate a statistically significant difference (P<0.05). The differences between the two groups were analyzed using an independent samples t-test.

^a^P<0.001, compared to baseline;

^b^P<0.001, compared to post-treatment (1st month). Inter-group differences were analyzed using repeated measures ANOVA. HDP, hypertonic dextrose prolotherapy; CPT, conventional physiotherapy; VAS, visual analog scale; WOMAC, Western Ontario and McMaster Osteoarthritis Index.

**Table III:** Baseline and after treatment (at 1 and 3 months) follow-up results of isokinetic parameters of the groups.

Parameter	HDP (n=30)	CPT (n=30)	P-value
	Mean ± SD		
Extensor PT			
60˚/sec AV (Nm)			
Baseline	43.37±16.63	39.63±17.49	0.400
After treatment			
1st month	53.10±17.06^a^	46.70±18.37^a^	0.167
3rd month	63.17±16.84^a,b^	54.67±16.89^a,b^	0.056
180˚/sec AV (Nm)			
Baseline	29.27±9.25	30.27±10.66	0.699
After treatment			
1st month	37.30±9.24^a^	39.57±12.32^a^	0.424
3rd month	47.73±10.55^a,b^	46.03±11.91^a,b^	0.561
Flexor PT			
60˚/sec AV. (Nm)			
Baseline	17.57±10.29	21.90±13.01	0.158
After treatment			
1st month	23.73±11.83^a^	28.50±15.99^a^	0.195
3rd month	32.27±15.38^a,b^	37.00±21.00^a,b^	0.324
180˚/sec AV. (Nm)			
Baseline	11.67±6.84	19.90±9.60	**0.001**
After treatment			
1st month	17.67±7.41^a^	28.80±12.61^a^	**0.001**
3rd month	25.77±10.08^a,b^	35.30±15.24^a,b^	**0.006**

Values in bold font indicate a statistically significant difference (P<0.05). The differences between the two groups were analyzed using an independent samples t-test.

^a^P<0.001, compared to baseline;

^b^P<0.001, compared to post-treatment (1st month). Inter-group differences were analyzed using repeated measures ANOVA. HDP, hypertonic dextrose prolotherapy; CPT, conventional physiotherapy; PT, peak torque; AV, angular velocity.

**Table IV:** Comparison of the differences between the scores of the groups.

Δ%	BT to AF (1 month)	BT to AF (3 months)	AF (1 month) to AF (3 months)
	Mean ± SD		
VAS for pain			
HDP	1.86±1.00	3.90±1.24	2.03±0.71
CPT	1.60±0.49	2.80±0.76	1.20±0.40
P-value	**0.001**	**0.001**	**0.001**
WOMAC			
HDP	4.06±1.66	7.90±2.29	3.83±2.18
CPT	2.50±0.86	4.761.43	2.26±1.17
P-value	**0.001**	**0.001**	**0.001**
Extensor PT, 60˚/sec AV			
HDP	-9.73±5.53	-19.80±8.03	-10.06±5.70
CPT	-7.06±3.05	-15.03±4.03	-7.96±3.32
P-value	**0.010**	**0.001**	**0.001**
Extensor PT, 180˚/sec AV			
HDP	-8.03±5.34	-18.46±8.07	-10.43±4.70
CPT	-9.30±3.71	-15.76±4.63	-6.46±2.52
P-values	0.202	**0.005**	**0.001**
Flexor PT, 60˚/sec AV			
HDP	-6.16±5.98	-14.70±9.91	-8.53±5.80
CPT	-6.60±5.28	-15.10±9.47	-8.50±5.52
P-value	0.930	0.128	0.911
Flexor PT, 180˚/sec AV			
HDP	-6.00±4.57	-14.10±6.94	-8.10±5.18
CPT	-8.90±5.09	-15.40±7.42	-6.50±3.97
P-value	**0.001**	**0.840**	**0.001**

Values in bold font indicate a statistically significant difference (P<0.05). HDP, hypertonic dextrose prolotherapy; CPT, conventional physiotherapy; VAS, visual analog scale; WOMAC, Western Ontario and McMaster Osteoarthritis Index; BT, before treatment; AT, after treatment; AV, angular velocity; PT, peak torque.

## Data Availability

The datasets used and/or analyzed during the current study are available from the corresponding author on reasonable request. The present study was registered in the ClinicalTrials.gov database (NCT04958213).
